# Efficacy of *Steinernema feltiae* Nematode Against Lesser Mealworm (*Alphitobius diaperinus*) Populations from Poultry Farms in Türkiye

**DOI:** 10.3390/vetsci11110567

**Published:** 2024-11-14

**Authors:** Burak Polat, Aysegul Cengiz, Samed Koc, Sevval Kahraman Kokten, Zeynep Nur Gultekin, Cansu Caliskan, Serap Kocaoglu Cenkci, Tolga Yildirim, Ozge Tufan-Cetin, Huseyin Cetin

**Affiliations:** 1 Department of Biology, Faculty of Science, Akdeniz University, Antalya 07070, Türkiye; bpolatant@gmail.com (B.P.); aysegulcengiz@akdeniz.edu.tr (A.C.); sevvaldilarakahraman@gmail.com (S.K.K.); zeynepnurgultekin@gmail.com (Z.N.G.); cansucaliskan0724@gmail.com (C.C.); tyildirim@akdeniz.edu.tr (T.Y.); 2 Laboratory Animals Application and Research Centre, Akdeniz University, Antalya 07070, Türkiye; samedkoc@akdeniz.edu.tr; 3 Department of Nutrition and Dietetics, Faculty of Health Sciences, Akdeniz University, Antalya 07070, Türkiye; skocaoglu@akdeniz.edu.tr; 4 Department of Environmental Protection Technology, Vocational School of Technical Sciences, Akdeniz University, Antalya 07070, Türkiye; ozgetufan@akdeniz.edu.tr

**Keywords:** biological control, entomopathogenic nematode, integrated pest management, larvicides, poultry pests, *Steinernema*

## Abstract

In the veterinary field, particularly in the poultry farming sector, *Alphitobius diaperinus* (Panzer, 1797) (Coleoptera: Tenebrionidae) is a significant pest that causes economic losses and acts as a vector for various pathogens. This study is the first to evaluate the efficacy of the entomopathogenic nematode *Steinernema feltiae* Filipjev, 1934 against both the larval (fifth instar) and imago (adult) of *A. diaperinus* from various Turkish populations. The insects were collected from six poultry farms in the provinces of Balikesir, Bolu, Canakkale, Manisa, and Izmir in Türkiye, bred, and then tested at concentrations ranging from 25 to 200 infective juveniles (IJs) per milliliter. Mortality was monitored over a five-day period. The findings revealed that larvae were much more susceptible to *S. feltiae* nematodes compared to adults, with LC_50_ values of larvae ranging from 33.17 to 83.12 IJs/mL. The Manisa population was the most susceptible, while the Izmir population was the most resistant. These results suggest that *S. feltiae* could be an effective biological control agent for managing *A. diaperinus* in Turkish poultry farms, reducing the reliance on chemical insecticides.

## Simple Summary

In poultry farms, a beetle called the lesser mealworm (*Alphitobius diaperinus*) is a common pest that causes significant problems. This beetle not only damages the structures where poultry are kept but also spreads harmful bacteria, such as *Salmonella* and *Escherichia*, which can cause diseases in poultry and pose risks to food safety. Traditionally, chemical insecticides have been used to control these pests, but they can leave harmful residues in poultry products and lead to pesticide resistance. Our study tested a natural method using a type of beneficial nematode, *Steinernema feltiae*, which is a microscopic worm that can kill the beetles without harming the poultry or leaving chemical residues. We found that these nematodes were particularly effective against beetle larvae. The results suggest that *S. feltiae* could be a valuable tool in reducing the need for chemical insecticides, making poultry farming safer and more sustainable. This natural approach can help protect animal health, reduce environmental impacts, and ensure safer food products for consumers.

## 1. Introduction

According to the Turkish Statistical Institute, Türkiye’s population grew by about 25%, from 67.8 million in 2000 to 85.4 million in 2023, significantly impacting animal and plant-based food production, as seen in many other countries. To meet the dietary needs, particularly protein, of the expanding population, there has been a notable rise in poultry production in recent years [1]. Furthermore, Türkiye has become a global leader in chicken meat production. According to data from the Turkish Poultry Meat Producers and Breeders Association, a total of 2,471,641 tons of poultry meat was produced in 2022, including 2,417,995 tons of chicken meat and 53,646 tons of turkey meat. The “Poultry Farming” report, published in 2023 by the Turkish Agricultural Economics and Policy Development Institute, reported that in 2020, Türkiye had 71 hatcheries, 2403 breeder houses, 13,018 broiler houses, and 4975 layer houses. The growing demand for chicken meat, due to its nutritional value, affordability, and export potential, suggests that the number of poultry farms in Türkiye will expand rapidly [2].

The poultry industry in Türkiye, comprising numerous businesses, faces challenges such as high operational costs (feed, electricity, labor), the need to maintain breeding facilities, and the prevention of animal diseases, all requiring substantial budgets and labor.

One of the most pressing issues that poultry farms face is the presence of harmful rodents, mites, and insects [3]. These pests can carry pathogens that cause diseases in poultry, lower productivity, and damage infrastructure, electrical systems, insulation, and the walls of buildings and facilities. Research worldwide indicates that chemical pesticides are primarily used on poultry farms to control these pests. However, it has been reported that on some farms, these pesticides have contaminated products like meat and eggs [4,5].

**Figure 1:**
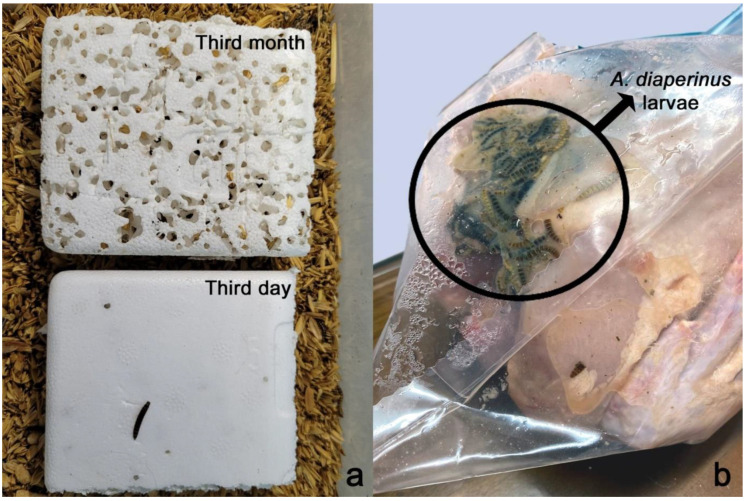
(**a**) Damage to polyurethane material caused by *Alphitobius diaperinus* larvae under laboratory conditions over time: noticeable degradation in third month compared to minimal damage on third day. (**b**) Presence of *A. diaperinus* larvae on packaged carcass intended for sale. (Images by Huseyin Cetin).

The lesser mealworm, or darkling beetle, *Alphitobius diaperinus* (Panzer, 1797) (Coleoptera: Tenebrionidae), is a common pest in poultry farming facilities. Materials used as poultry house litter, such as rice husks, along with moist chicken droppings (chicken manure) and feathers that accumulate on them, create an ideal environment for the growth of *A. diaperinus*. This pest serves as a carrier and/or reservoir for pathogens that cause a range of diseases, including bacteria (such as *Streptococcus*, *Escherichia*, and *Salmonella*), viruses (responsible for diseases like Marek’s disease and Newcastle disease), fungi, and certain helminths (e.g., *Hadjelia truncate*, *Heterakis gallinarum*) [6,7,8]. It leads to significant economic losses by damaging insulation systems in poultry facilities. Larvae burrow into the wood and polyurethane panels used for constructing the inner walls of poultry facilities, causing severe damage that can nearly destroy these materials within a few years. Figure 1a shows the damage to polyurethane material caused by litter beetle larvae under laboratory conditions within three months. Additionally, lesser mealworms can be ingested by birds into their crops (craws) while pecking at the litter and may remain there until slaughter. After slaughter, these insects can still be found in the craw of processed carcasses, negatively affecting the product’s quality (Figure 1b).

While there are some variations in how poultry farms manage *A. diaperinus* control, many farms apply both larvicides (such as diflubenzuron or pyriproxyfen) and adulticides (such as alphacypermethrin, β-cyfluthrin, deltamethrin, lambda-cyhalothrin, or permethrin) to the chicken manure and organic waste left on the poultry house floor after the chickens have completed their growth and left the coop [9,10]. In some cases, the manure is removed from the poultry house without the use of insecticides. The poultry house is then cleaned and disinfected, a mixture of larvicide and adulticide is applied to the floor-side walls, ensuring it penetrates cracks–crevices. Following this, insect growth regulators are often applied to the litter material on the floor before the chicks are introduced to the poultry farm.

Although efforts to control *A*. *diaperinus* using synthetic insecticides have been largely successful in Türkiye, poultry farmers and biocidal product applicators report that these insecticides are sometimes not sufficiently effective (personal communication). However, in Türkiye, there are no documented cases of entomopathogenic viruses and fungi, nor is there any research on insecticide resistance. Similarly, research conducted in many parts of the world has found that litter beetles are developing varying levels of resistance to these products. Moreover, the potential residue from synthetic chemicals in chicken meat remains a significant concern [11,12].

In integrated pest management, one of the most effective methods reported to prevent the issues of chemical insecticide residues and resistance development in the fight against *A. diaperinus* is the use of entomopathogenic nematodes (EPNs) [13]. To date, approximately 100 species of EPNs have been identified, with about 30 species belonging to the genus *Steinernema* in the family Steinernematidae. *Steinernema feltiae* Filipjev, 1934, a species of EPN, is a well-known biological control agent used against a variety of insect pests, including many from the orders Lepidoptera and Coleoptera. EPNs have been proven to be effective against numerous agricultural and forest pests from these orders, providing an eco-friendly alternative to chemical pesticides [14,15,16,17]. It is known that insect deaths begin to appear approximately 48–72 h after the nematode infects the host’s body. In this study, we aimed to investigate the invasiveness of *S. feltiae* on both larval and adult stages of six populations sampled from various provinces of Türkiye. To date, no research has been conducted in Türkiye on the use of this nematode species for controlling lesser mealworms.

## 2. Materials and Methods

### 2.1. Tested Insects

**Figure 2:**
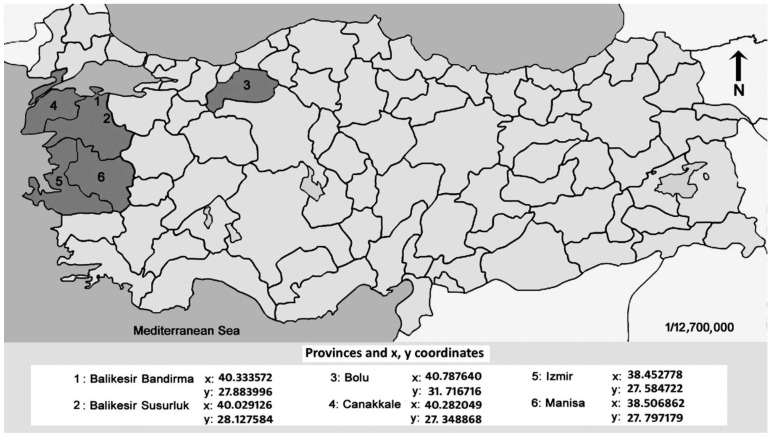
GPS coordinates of sites where *Alphitobius diaperinus* larvae and adults were collected in Türkiye.

The adults and larvae of *A. diaperinus* were collected from the organic matter on the floor and near the walls of six broiler chicken poultry farms located in Balikesir, Bolu, Canakkale, Izmir, and Manisa provinces in Türkiye between June and August 2021 to establish laboratory colonies for testing. Sampling was conducted in provinces that house approximately 75% of Türkiye’s broiler farms. The GPS coordinates of the collection sites are provided in Figure 2. Beetle adults and larvae were transported to the Faculty of Science, the Department of Biology at Akdeniz University, and placed in the Vector Ecology and Control Laboratory within 48 h of collection. Insects were reared in plastic containers (20 × 30 × 40 cm) with a capacity for over 250 individuals, using a daily diet consisting of layer chicken feed and fish food. The rearing medium was changed monthly, and an adequate amount of water was provided to the insects by soaking a cotton ball. The moisture level of the cotton was monitored daily, and water was replenished every two days to compensate for evaporation and consumption. Insects were kept at a temperature of 24 ± 2 °C and 50 ± 10% RH in incubators with a photoperiod of 12:12 (light/dark) h.

### 2.2. Tested Nematodes

The commercial product Nematac, which consists of the EPN *S. feltiae* (purchased from Bioglobal Zirai Biyolojik Sistemler Tar. Dan. Gid. Tar. San. ve Tic. A.S., Bahçelievler Mah. Konyaalti Cad. No: 54/13 Muratpasa, Antalya, Türkiye), was used in the experiments. The nematodes were stored at 4 °C and used within one week.

### 2.3. Nematode Efficacy Testing on Lesser Mealworms

Biological efficacy tests were conducted using fifth-generation (F5) lesser mealworms, which were placed in sterile Petri dishes (Nunc, 90 mm diameter × 15 mm height, surface area 58 cm^2^, vented). Each dish contained 10 L5 larvae (approximately 25 days old) or mixed-gender adult beetles (10–15 days old). Both life stages were exposed to varying rates of infective juveniles (IJs) of *S. feltiae* in distilled water, with rates of 25, 50, 100, and 200 IJs per milliliter (equivalent to 2.5, 5, 10, and 20 nematodes per beetle host) being applied to filter paper (56.7 cm^2^) within the dishes. These rates were selected based on findings from an earlier study [13]. To prevent the reduction in moisture levels in the Petri dishes and to avoid the drying of the filter papers, 0.5 mL of distilled water was added to the filter papers from the edge of the Petri dish using an insulin syringe on days 2, 3, and 4. Mortality was monitored every 24 h over a period of five days. Control groups received only distilled water. The experiments were conducted under controlled conditions of 24 ± 2 °C and 50 ± 10% relative humidity, with a photoperiod of 12:12 (light/dark) hours. Each treatment, including controls, was repeated at least three times. Adults and larvae were considered dead if they showed no movement after being prodded (for 1 s, three times). At the end of the experiments, on the 5th day, the presence of nematodes in the dead insects was confirmed through dissection.

### 2.4. Statistical Analysis

Mortality data were corrected using Abbott’s formula when control mortality ranged from 5% to 20%; no correction was applied if control mortality was less than 5% [17]. LC_50_ and LC_90_ values were then calculated through probit analysis using SPSS 20 software [18]. Statistical differences between the data were assessed using one-way ANOVA, and the groups responsible for these differences were identified with Duncan’s new multiple range test, both performed with SPSS version 20 software.

## 3. Results

The lethal effects of the *S. feltiae* nematode on both the larvae and adult stages of the lesser mealworm, *A. diaperinus*, were investigated across various populations. LC_50_ and LC_90_ values for adults could not be calculated because the data for adult insects were below the required range for probit analysis. However, the data obtained from larvae fell within the range suitable for probit analysis, and the results are presented in Table 1.

The susceptibility of different populations to *S. feltiae* varied significantly, allowing us to rank them from most susceptible to most durable based on their LC_50_ and LC_90_ values. The Manisa strain was the most susceptible, with an LC_50_ of 34.25 IJs/mL and an LC_90_ of 93.52 IJs/mL. In contrast, the Izmir strain was the most resistant, with an LC_50_ of 83.12 IJs/mL and an LC_90_ of 183.06 IJs/mL. The other populations fell in between, with varying degrees of susceptibility (Table 1).

**Figure 3:**
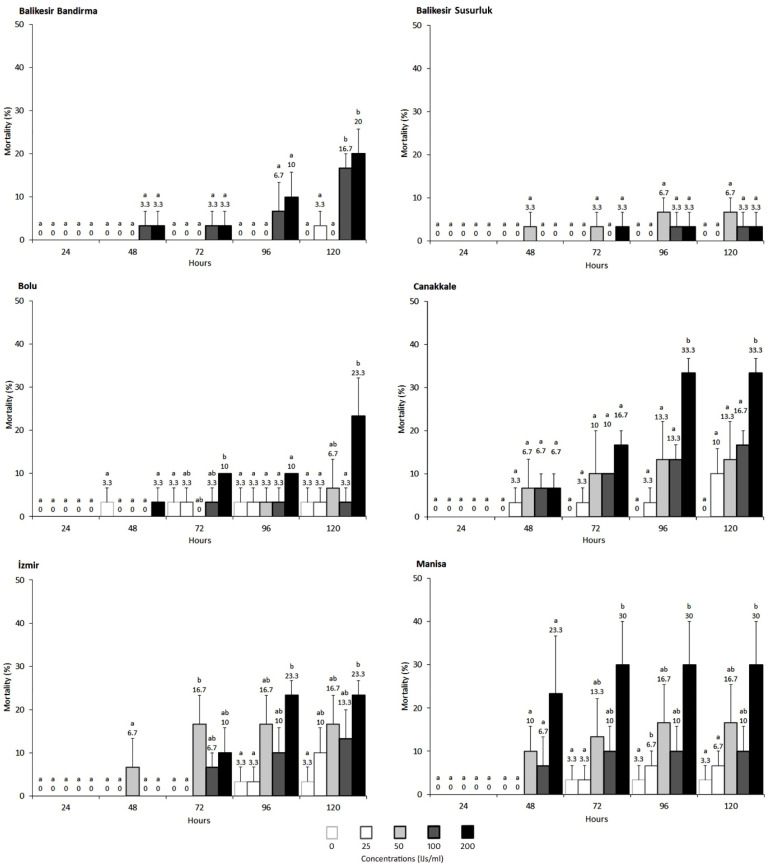
The lethal effects of the nematode *Steinernema feltiae* on different populations of adult *Alphitobius diaperinus* from various provinces of Türkiye. Percent mortality averages were statistically compared for each strain using Duncan’s new multiple range test (*p* ≤ 0.05). If the lower case letters are the same, there is no statistical difference.

**Figure 4:**
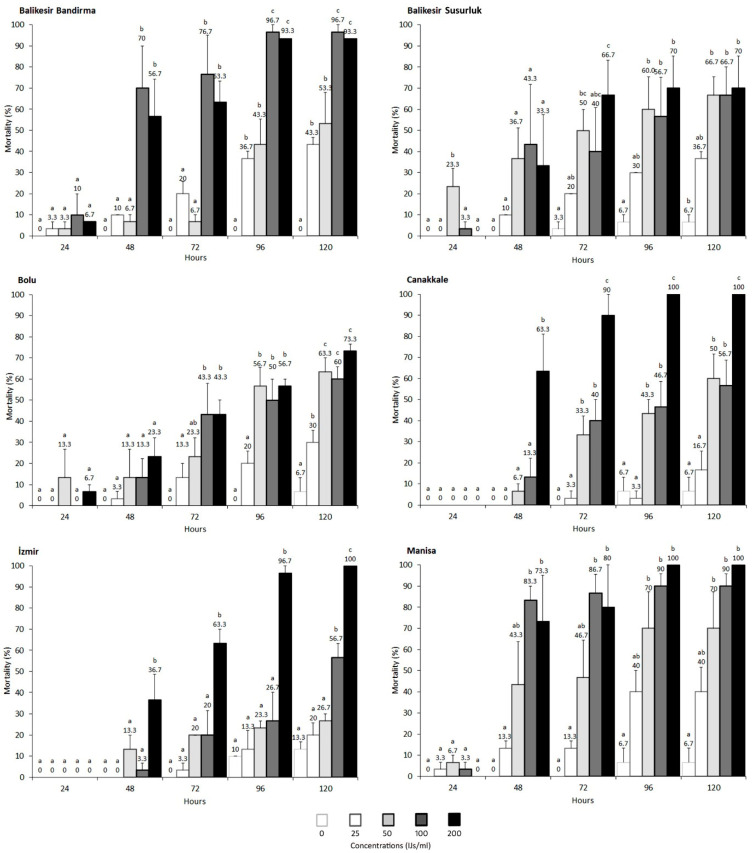
The lethal effects of the nematode *Steinernema feltiae* on different populations of larvae *Alphitobius diaperinus* from various provinces of Türkiye. Percent mortality averages were statistically compared for each strain using Duncan’s new multiple range test (*p* ≤ 0.05). If the lower case letters are the same, there is no statistical difference.

When examining the lethal effects on the Canakkale strain, adult mortality ranged from 10% to 33% at the tested concentrations after 120 h, while larvae mortality ranged from 16% to 100%. The LC_50_ and LC_90_ values for the larvae were 59.95 IJs/mL and 168.47 IJs/mL, respectively. The nematode’s lethal effect was significantly higher in larvae at concentrations of 50, 100, and 200 IJs/mL, with mortality beginning to rise notably after 72 h (Table 1, Figure 3 and Figure 4).

In the Balikesir Bandirma strain, adult mortality peaked at 20% at the highest concentration of 200 IJs/mL, while larvae mortality reached 96.67%. The larvae were consistently more sensitive than adults, with LC_50_ and LC_90_ values of 33.17 IJs/mL and 122.12 IJs/mL, respectively. Mortality in adults ranged from 0% to 20%, compared to 43% to 93% in larvae after 120 h (Table 1, Figure 3 and Figure 4).

The susceptibility of the Balikesir Susurluk strain revealed that adult beetles were largely unaffected by the nematodes, whereas larvae experienced mortality rates between 36% and 70% after 120 h. The LC_50_ was 44.44 IJs/mL, and the LC_90_ was 1073.14 IJs/mL. Larvae mortality began to increase noticeably at 48-72 h (Table 1, Figure 3 and Figure 4).

In the Izmir strain, adult mortality after 120 h ranged from 10% to 23%, while larvae mortality ranged from 20% to 100%. The LC_50_ and LC_90_ values for the larvae were updated to 83.12 IJs/mL and 183.06 IJs/mL, respectively. Larvae were particularly susceptible at concentrations of 100 and 200 IJs/mL.

For the Manisa strain, adult mortality was minimal, with a maximum of 6% observed at the tested concentrations. Larvae mortality was slightly lower than in other populations, ranging from 36% to 70% after 120 h. The LC_50_ was calculated to be 34.25 IJs/mL, and the LC_90_ was 93.52 IJs/mL (Table 1, Figure 3 and Figure 4).

In the Bolu strain, adult mortality reached a maximum of 23% at the highest concentration. Mortality rates in larvae, however, were higher, ranging from 30% to 73%. The LC_50_ and LC_90_ values for the larvae were calculated to be 58.86 IJs/mL and 708.21 IJs/mL, respectively (Table 1, Figure 3 and Figure 4).

Based on the LC_50_ values obtained from larvae, the most susceptible strain to *S. feltiae* was from Balikesir Bandirma (LC_50_ = 33.17 IJs/mL), followed closely by the Manisa strain (LC_50_ = 34.25 IJs/mL). The Balikesir Susurluk strain (LC_50_ = 44.44 IJs/mL), Bolu strain (LC_50_ = 58.86 IJs/mL), and Canakkale strain (LC_50_ = 59.95 IJs/mL) showed intermediate levels of susceptibility. The Izmir strain (LC_50_ = 83.12 IJs/mL) was the most resistant.

In terms of the LC_90_ values also obtained from larvae, the Manisa strain (LC_90_ = 93.52 IJs/mL) exhibited the lowest value, indicating relatively high susceptibility, while the Balikesir Susurluk strain (LC_90_ = 1073.14 IJs/mL) demonstrated the highest level of resistance. The other strains had LC_90_ values ranging between 122.12 IJs/mL (Balikesir Bandirma) and 708.21 IJs/mL (Bolu), further emphasizing the variation in resistance levels.

## 4. Discussion

The litter beetles examined in our study are a species that transmit bacteria to chicken populations in poultry houses. Barua et al. [19] reported that *Salmonella enteritidis* and *Campylobacter jejuni* were transmitted to new flocks in successive breeding cycles through litter beetles and litter left on the ground. After exposing adult and larval litter beetles to two different concentrations of fluorescently labeled *Salmonella enterica* over various time intervals within an hour, it was observed that the bacteria commonly passed through the beetle’s gut. This allowed the beetles to spread viable pathogenic bacteria within 2–3 h [20]. In addition to being vectors of pathogens, lesser mealworms pose a significant risk to food safety through pesticide residues from the chemicals used in their control, which can accumulate in the poultry environment and ultimately contaminate chicken products.

One of the most significant issues in recent decades has been pesticide residues in food products [21]. As the global population increases, the use of pesticides has unfortunately become necessary to produce enough food of adequate quality. In this context, chicken meat, due to its high nutritional value and lower cost compared to red meat, has become a staple food. However, chickens are often exposed to pesticides in the environments where they are raised, slaughtered, and packaged [22]. The fact that thousands of chickens are kept together in poultry farms makes pest control more challenging. Consequently, effective insect and mite control in poultry houses can typically only be achieved when the house is emptied and cleaned. The primary reason for this is the potential toxic effects of the chemicals on the animals and the problem of residue [4,5]. Luckmann et al. [23] conducted a study on *Gallus domesticus* (L. 1758) embryos, revealing that exposure to Pyriproxyfen—a larvicide commonly used on farms to control lesser mealworms—causes DNA damage, impairs cell proliferation, and induces apoptosis in the layers of brain vesicles. They reported that sublethal doses of Pyriproxyfen are a potent stressor for neurodevelopment, significantly damaging the architecture of brain vesicles. In this regard, in addition to the chemicals applied after cleaning the poultry house, it would be beneficial to apply EPNs—biologically very safe organisms—to the damp corners of the house as part of integrated pest management efforts, even while the chickens are still present.

Although chemical insecticides and acaricides are highly effective and predominantly used for controlling lesser mealworms in poultry farms, their toxicological risks require careful handling, leading to a significant increase in research on the usability of biological control agents in recent years. Among the entomopathogenic bacteria, viruses, fungi, and nematodes used in biological control against various pests, nematodes have been proven to be the most effective against lesser mealworms [24]. For instance, in a study conducted by Koc et al. [25], where five different species of *Bacillus* bacteria were tested against lesser mealworms, it was found that the bacterial toxins did not exhibit sufficient control activity against the beetles. Furthermore, EPNs present a significant advantage in that they do not cause residue problems, and the possibility of insects developing resistance is very low, almost impossible. Typically, once a sufficient number of nematodes enter the host’s body, death occurs within a few days. After entering and killing the target organism, they can reproduce and move on to infect other pests in moist environments [26,27]. This makes them particularly beneficial in poultry house floors where lesser mealworms thrive, as the nematodes can spread from one beetle to another. Moreover, the moisture from animal waste and water from drinkers can enhance the survival of these nematodes. Geden et al. [26] studied the application of *S. feltiae* to the soil floors of one broiler and two turkey houses. They found that in all three farms, insect populations in the treated houses grew more slowly compared to untreated houses. Additionally, when soil samples from the farm floors were tested by introducing new insect larvae, it was observed that the nematodes remained active in the environment for 3–7 weeks. It is also noted that temperature is another influential factor in the persistence of nematodes in the environment. In soil maintained at temperatures above 24 °C, no nematodes survived beyond 2 weeks after treatment. However, nematodes were detected up to 9 weeks after treatment in soil maintained at 20 °C and 24 °C [28].

To date, limited research has been conducted on controlling lesser mealworms using *S. feltiae*. Szalanski et al. [29] tested the infectivity of 12 different populations from three *Steinernema* species (*S. carpocapsae*, *S. feltiae*, and *S. scapterisci*) against adult *A. diaperinus*. Among the five most promising populations, LC_50_ values ranged from 1.5 to 77.0 nematodes per host in filter paper assays. In poultry litter material, the LC_50_ values had been calculated as 5.8 nematodes per host for the Mexican *S. carpocapsae* strain and 14.6 nematodes per host for the Pye *S. feltiae* strain.

Our research findings clearly indicate that the larvae of the six lesser mealworm populations are more susceptible to *S. feltiae* compared to the adults. This observation has also been documented in previous studies by other researchers with different nematode species. For example, Koc et al. [13] examined the effectiveness of *S. carpocapsae* on four Turkish lesser mealworm populations (Manisa, Izmir, Canakkale, and Balikesir). Their study revealed that larvae were more susceptible to the nematode compared to adults. Among the populations, Balikesir was the most susceptible, with LC_50_ values of 85.9 IJs/mL for adults and 31.2 IJs/mL for larvae, while the Manisa strain was the most resistant, with LC_50_ values of 418.8 IJs/mL for adults and 70.9 IJs/mL for larvae. When examining the study by Del Valle et al. [30] on the lethality of the nematodes *S. rarum* and *Heterorhabditis bacteriophora* toward lesser mealworms, it was reported that adult beetles were more resistant to the lethal effects of both nematodes compared to larvae. Alves et al. [31] also found that adults were less susceptible than larvae to *S. glaseri* and *S. carpocapsae*. This finding is due to several main reasons. Firstly, adults are generally smaller in size and possess a harder and more durable exoskeleton with fewer folds, making it more difficult for the nematodes to penetrate their bodies. In contrast, larvae have a body that is segmented in many parts and longer in structure, making them more susceptible to nematode infection. In integrated pest management, it is recommended to prioritize larval control before targeting adult pests, with EPNs emphasized as an excellent and effective choice for this purpose.

Compared to the study by Koc et al. [13] on lesser mealworm populations from some of our locations, similar susceptibilities were observed in three out of four populations tested in both studies, indicating consistent responses of the insect to *S. carpocapsae*. When comparing the LC_50_ values between the two nematode species, notable differences emerge. The Manisa strain shows a significantly higher LC_50_ value with *S. carpocapsae* (70.9 IJs/mL) compared to *S. feltiae* (34.25 IJs/mL), indicating a higher resistance to *S. carpocapsae*. The Izmir strain, however, exhibits a lower LC_50_ with *S. carpocapsae* (34.8 IJs/mL) compared to *S. feltiae* (83.12 IJs/mL), suggesting greater susceptibility to *S. carpocapsae*. In contrast, the Balikesir Bandirma strain’s LC_50_ values are quite similar between the two nematode species, with 31.2 IJs/mL for *S. carpocapsae* and 33.17 IJs/mL for *S. feltiae*. The Canakkale strain shows a slightly lower LC_50_ with *S. carpocapsae* (39.8 IJs/mL) compared to *S. feltiae* (59.95 IJs/mL), suggesting greater susceptibility to *S. carpocapsae*. Overall, the susceptibility of the populations to *S. carpocapsae* and *S. feltiae* varies depending on the strain. While some populations, like the Izmir and Canakkale populations, are more susceptible to *S. carpocapsae*, others, like the Manisa strain, show greater susceptibility to *S. feltiae*. This indicates that the effectiveness of each nematode species as a larvicide depends on the specific strain being targeted.

In our research, we used the contact method (Petri dish) to evaluate the effects of nematodes on *A. diaperinus*. Although some studies suggest that the feeding method, particularly involving nematodes present in infected cadavers, may be more effective [32], there are significant challenges associated with using the feeding method. One concern is that digestive enzymes in the insect gut could potentially harm the nematodes during ingestion, reducing their effectiveness. Additionally, in the environments where these insects live, there is a wide variety of materials that may be preferred as food, introducing uncertainty as to whether the insects would consume the nematodes in sufficient quantities. The feeding method also introduces additional steps, such as the removal of infected cadavers from food remnants, which increases the risk of contamination [33]. Moreover, identifying a food composition that would both attract the insects and support the nematodes requires further research. In contrast, the contact method is more straightforward and widely used due to its simplicity and reliability. It allows nematodes to enter the insect host through multiple routes, such as the mouth, anus, and respiratory openings, making it a more direct and reliable method for ensuring nematode entry. This approach also allows for standardized exposure across all test subjects, helping to focus on the impact of different nematode concentrations in a controlled manner.

## 5. Conclusions

According to the results of this study, the entomopathogenic nematode *S. feltiae* was highly effective against the larval stage of *A. diaperinus* across different populations in Türkiye, with larvae showing significantly higher susceptibility compared to adults. The success of entomopathogenic nematodes in controlling *A. diaperinus* could help address concerns about pesticide residues and resistance by reducing reliance on chemical insecticides. Our findings suggest that integrating *S. feltiae* into pest management strategies in poultry farms may reduce the use of chemicals in poultry production systems, thereby enhancing both environmental safety and food quality. Future research should focus on the long-term viability of nematodes in poultry environments, their interactions with other pest control methods, and strategies to increase their lethal effects on adult insects.

## Figures and Tables

**Table 1:** LC_50_ and LC_90_ values (IJs/mL) of *Steinernema feltiae* against larvae of *Alphitobius diaperinus* for 120 h in different populations from Türkiye.

Strain	LC_50_	LC_90_	Χ^2^	*p*
Balikesir Bandirma	33.17	122.12	18.97	0.0001
Manisa	34.25	93.52	1.90	0.385
Balikesir Susurluk	44.44	1073.14	10.58	0.005
Bolu	58.86	708.21	11.59	0.003
Canakkale	59.95	168.47	39.67	0.0001
Izmir	83.12	183.06	26.39	0.0001

## Data Availability

The data presented in this study are available within the article’s figures and tables.
